# Supplementary material for: Nr4a1 suppresses cocaine-induced behavior via epigenetic regulation of homeostatic target genes
Source: Nat Commun. 2020 Jan 24;11:504. doi: 10.1038/s41467-020-14331-y (PMC6981219; doi:10.1038/s41467-020-14331-y)
Supplement: Supplementary file 1 — Supplementary Information [file 41467_2020_14331_MOESM1_ESM.pdf]

Cover Page – Supplementary Information

Nr4a1 suppresses cocaine-induced behavior via epigenetic regulation of homeostatic target genes

Carpenter M.D., et al.

Supplementary Figure 1

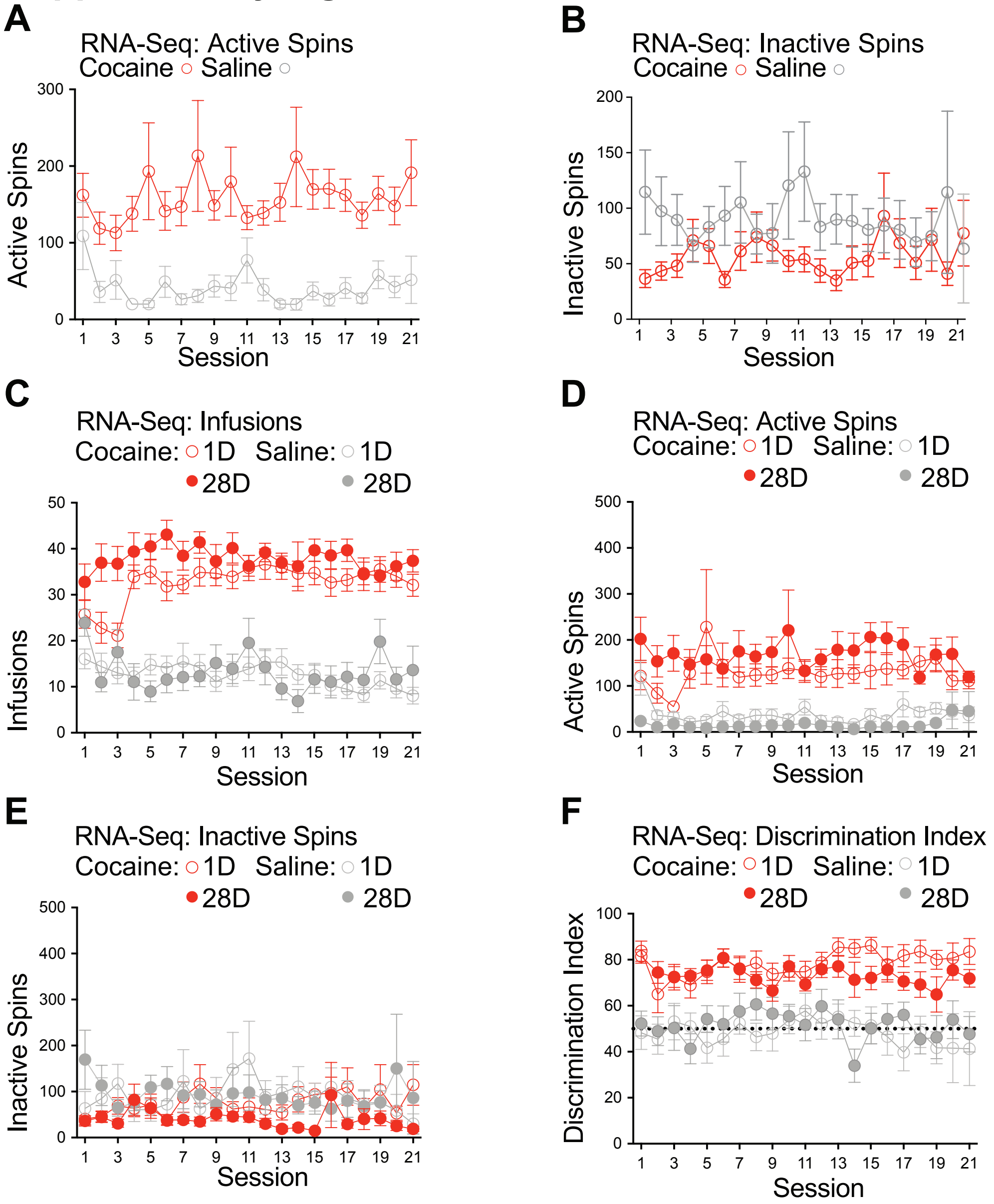

Supplementary Figure 1. Cocaine self-administration behavior RNA-Seq cohort. A. Cocaine self-administering mice responded significantly higher than saline self-administering mice (n=24 mice/group, two-way repeated measures ANOVA). *P<0.05. **B.** There were no significant differences in inactive spins (n=24 mice/group, two-way repeated measures ANOVA). *P<0.05. **C.** 1- and 28-days cocaine self-administering mice infused similar amounts of drug (n=24 mice/group, two-way repeated measures ANOVA, followed by Bonferroni's multiple comparisons test). *P<0.05. **D.** 1- and 28-days cocaine self-administering mice responded similar across sessions (n=24 mice/group, two-way repeated measures, followed by Bonferroni's multiple comparisons test). *P<0.05. **E.** 1- and 28-days cocaine self-administering mice responded similar on inactive spins across sessions (n=24 mice/group, two-way repeated measures ANOVA, followed by Bonferroni's multiple comparisons test). *P<0.05. **F.** 1- and 28-days cocaine self-administering mice discriminate between inactive and active spins across sessions (n=24 mice/group, two-way repeated measures ANOVA, followed by Bonferroni's multiple comparisons test). All error bars represent s.e.m. Source data and statistics provided as a Source Data file.

Supplementary Figure 2

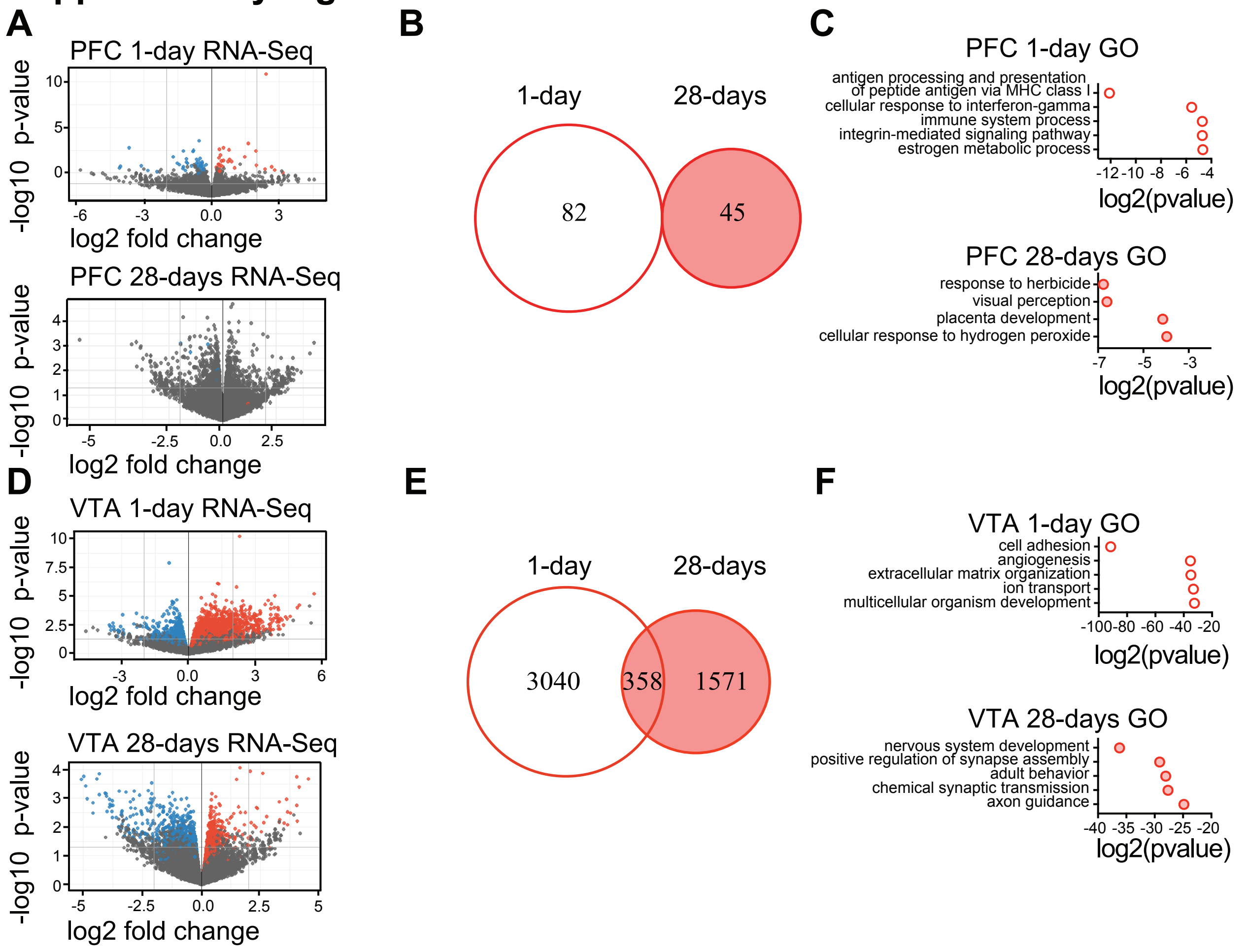

Supplementary Figure 2. PFC and VTA transcriptomics following 1- and 28-days of abstinence. A. Mice were sacrificed, and PFC collected at 1- and 28-days of abstinence and subjected to RNA-seq. Volcano plot showing differentially expressed genes (DEGs), significantly downregulated (Blue) and upregulated (red), q-value < 0.01. **B.** Comparison of DEGs at 1- and 28-days of abstinence, showing fewer .DEGs at 28-days of abstinence compared to 1-day, with no overlapping genes. **C.** Gene Ontology (GO) analysis of DEGs at 1-day (Top) and 28-days (Bottom) of abstinence. DEGs cluster in GO biological process of antigen processing and presentation (1-day) and estrogen metabolic process (28-day) **D.** Mice were sacrificed and VTA collected at 1- and 28-days of abstinence and subjected to RNA-seq. Volcano plot showing differentially expressed genes (DEGs), significantly downregulated (Blue) and upregulated (red), q-value < 0.05. **E.** Comparison of DEGs at 1- and 28-days of abstinence, showing a greater number of DEGs at 28-days of abstinence compared to 1-day, with few overlapping genes. **F.** Gene Ontology (GO) analysis of DEGs at 1-day (Top) and 28-days (Bottom) of abstinence. DEGs cluster in GO biological process of cell adhesion (1-day) and nervous system development (28-day) *P<0.05. All error bars represent s.e.m. Source data and statistics provided as a Source Data file.

Supplementary Figure 3

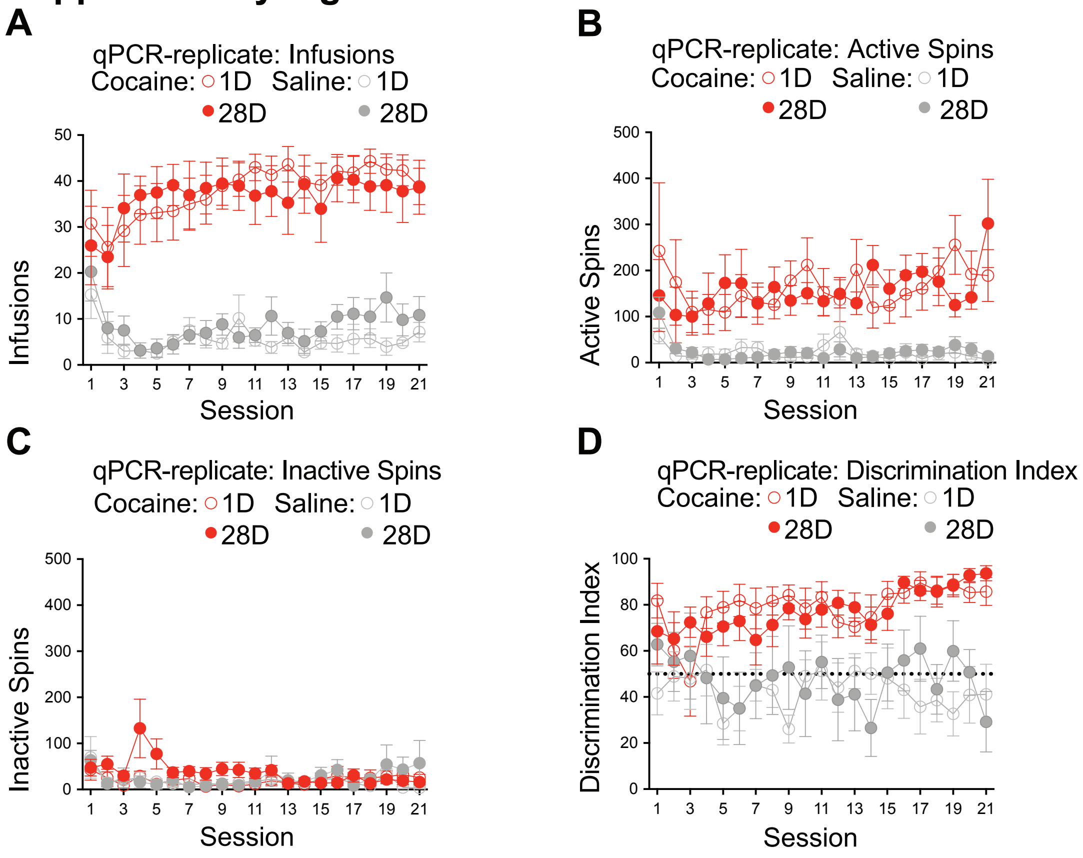

Supplementary Figure 3. Behavior data of RNA-Seq replication cohort. A. DEGs at 1-day of abstinence following cocaine SA measured by RNA-seq log2 fold change normalized to saline controls, q-value < 0.01 **B.** Validation of DEGs at 1-day of abstinence by qPCR in a biological replicate following cocaine SA (n=6, unpaired two-tailed *t* test, *Fmo2* P=0.002, *Ccdc141* P=0.0326, *Mertk* P=0.0048, *Nr4a3* P=0.0009, *Kirrel2* P=0.0179, *P<0.05), relative to saline controls (grey circles). *P<0.05. **C.** DEGs at 28-day of abstinence following cocaine SA measured by RNA-seq log2 fold change normalized to saline controls, q-value < 0.01 **D.** Validation of DEGs at 28-day of abstinence by qPCR in a biological replicate following cocaine SA (n=6, unpaired two-tailed *t* test, *E2f1* P=0.0136, *Mark2* P=0.0163, *Ndn* P=0.0235, *Robo* P=0.013, *P<0.05), relative to saline controls (grey circles *P<0.05.). All error bars represent s.e.m. Source data and statistics provided as a Source Data file.

Supplementary Figure 4

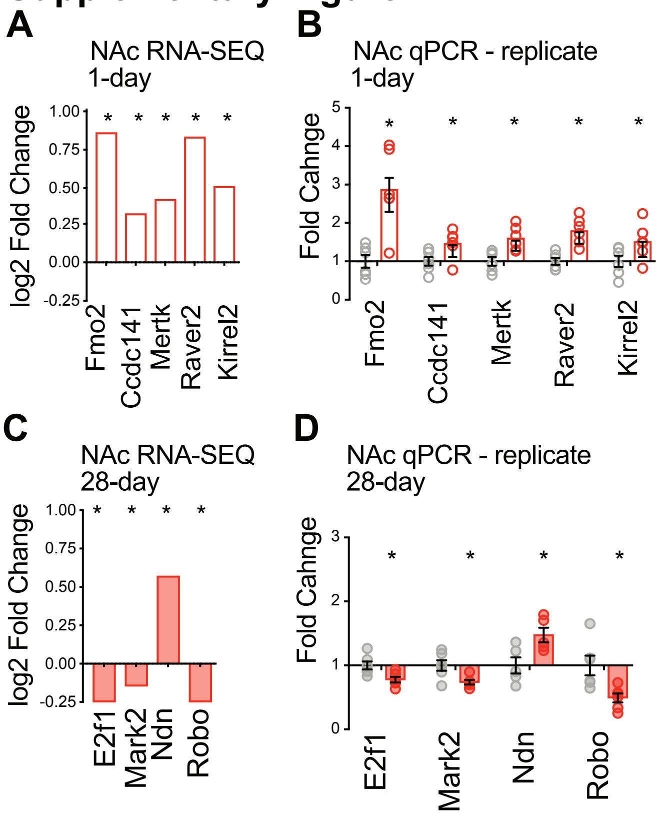

Supplementary Figure 4. qPCR validation of RNA-Seq **A.** DEGs at 1-day of abstinence following cocaine SA measured by RNA-seq log2 fold change normalized to saline controls, q-value < 0.01 **B.** Validation of DEGs at 1-day of abstinence by qPCR in a biological replicate following cocaine SA (n = 6, unpaired two-tailed t test, Fmo2 P=0.002, Ccdc141 P=0.0326, Mertk P=0.0048, Nr4a3 P=0.0009, Kirrel2 P=0.0179, *P<0.05), relative to saline controls (grey circles). *P<0.05. **C.** DEGs at 28-day of abstinence following cocaine SA measured by RNA-seq log2 fold change normalized to saline controls, q-value < 0.01 **D.** Validation of DEGs at 28-day of abstinence by qPCR in a biological replicate following cocaine SA (n = 6, unpaired two-tailed t test, E2f1 P=0.0136, Mark2 P=0.0163, Ndn P=0.0235, Robo P=0.013, *P<0.05), relative to saline controls (grey circles). *P<0.05. All error bars represent s.e.m. Source data and statistics provided as a Source Data file.

Supplementary Figure 5

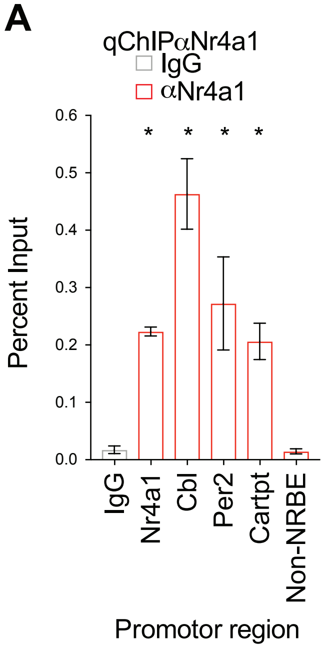

Supplementary Figure 5. Nr4a1 binding in promoter regions of genes differentially expressed at 1-day and 28-days of abstinence. A. Nr4a1 was enriched at the *Nr4a1, Cbl, Per2,* and *Cartpt,* promoters (One-way ANOVA; *Nr4a1* P=0.0114*, Cbl* P<0.0001*, Per2* P= 0.0031*, Cartpt* P=0.0188) but not at distal sites (Distal P>0.9999), normalized to percent input, when compared to IgG controls, *P<0.05. All error bars represent s.e.m. Source data and statistics provided as a Source Data file.

Supplementary Figure 6

A

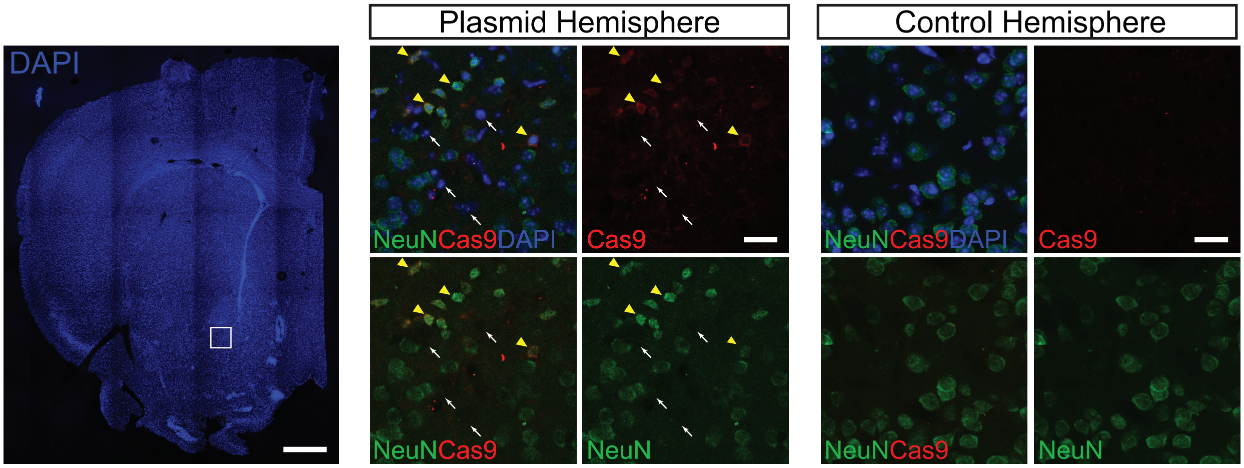

Magnification of boxed area
Scale bar = 500um

Plasmid hemisphere (left) and Control hemisphere (right).
Yellow arrow heads indicate NeuN+/Cas9+ cells
White arrows indicate NeuN-/Cas9- cells
Scale bar = 20um

Supplementary Figure 6. dCas9, NeuN+ and DAPI IHC in the NAc. A. dCas9-VP64 colocalizes with NeuN+ cells in the NAc. Plasmid hemisphere (right) transfected with dCas9 alone and control hemisphere transfected with reagent alone. Yellow arrow heads indicate NeuN+/dCas9-VP64+ cells. White arrows indicate NeuN-/Cas9- cells. DAPI = Blue, dCas9 = Red, NeuN=Green.

Supplementary Figure 7

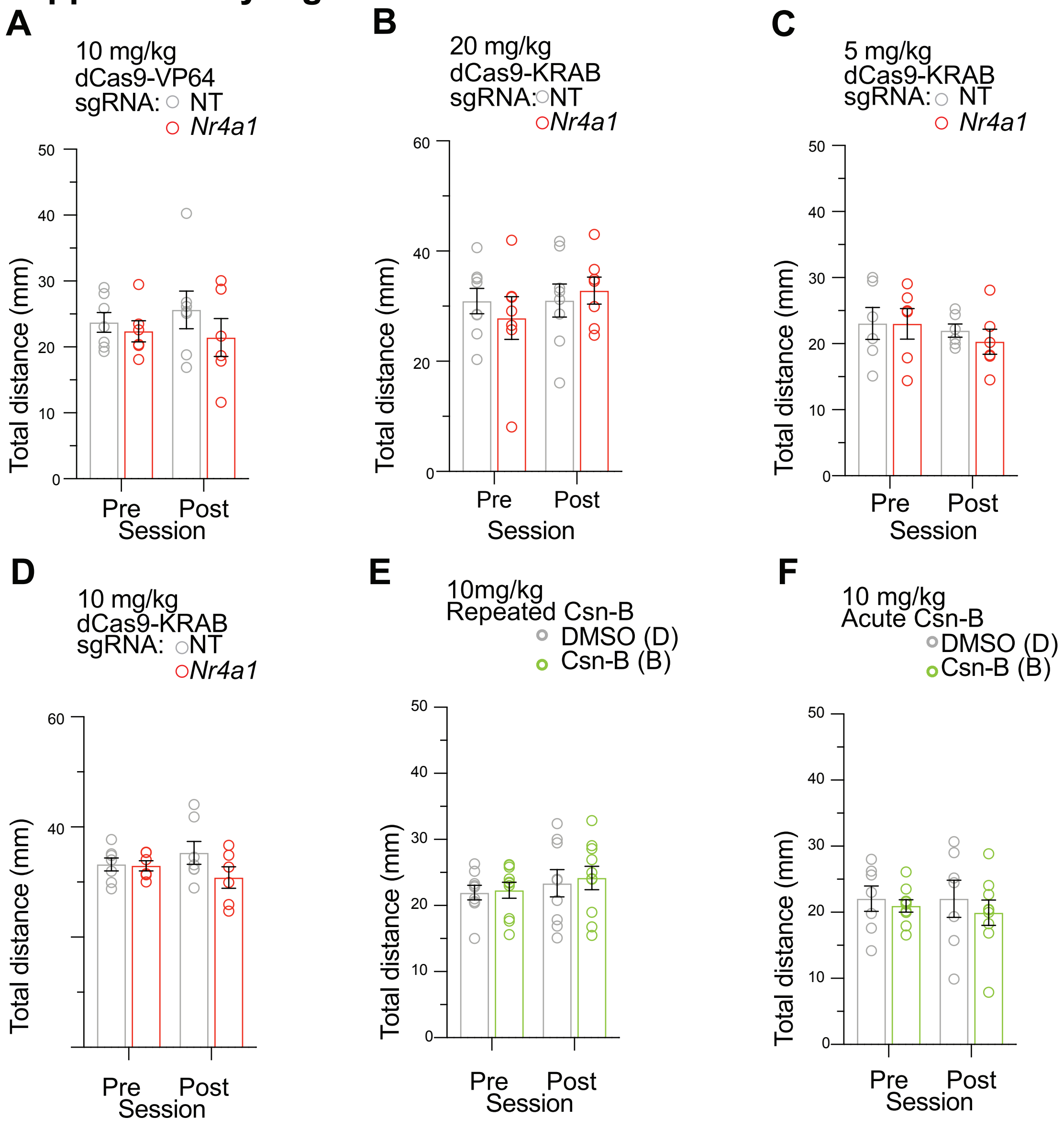

Supplementary Figure 7. Total distance traveled in cocaine CPP A. There were no significant differences in distance traveled when comparing sgRNA-*Nr4a1* to control NT at pretest and posttest (10 mg/kg, n=7 mice/group, two-way repeated measures ANOVA), *P<0.05. **B.** There were no significant differences in distance traveled when comparing sgRNA-*Nr4a1* to control NT at pretest and posttest when comparing sgRNA-*Nr4a1* to control NT (20 mg/kg, n=7 mice/group, two-way repeated measures ANOVA, *P<0.05. **C.** There were no significant differences in distance traveled when comparing sgRNA-Nr4a1 to control NT at pretest and posttest (5 mg/kg, n=7 mice/group, two-way repeated measures ANOVA, *P<0.05. **D.** There were no significant differences in distance traveled when comparing sgRNA-231 to control NT at pretest and posttest (10 mg/kg, n=7 mice/group, two-way repeated measures ANOVA, *P<0.05. **E.** There were no significant differences in distance traveled when comparing Csn-B to control DMSO at pretest and posttest (repeated Csn-B, n=9 mice/group, two-way repeated measures ANOVA, *P<0.05. **F.** There were no significant differences in distance traveled when comparing Csn-B to control DMSO at pretest and posttest (10 mg/kg, n=8 mice/group, two-way repeated measures ANOVA, *P<0.05. Source data and statistics provided as a Source Data file.

Supplementary Figure 8

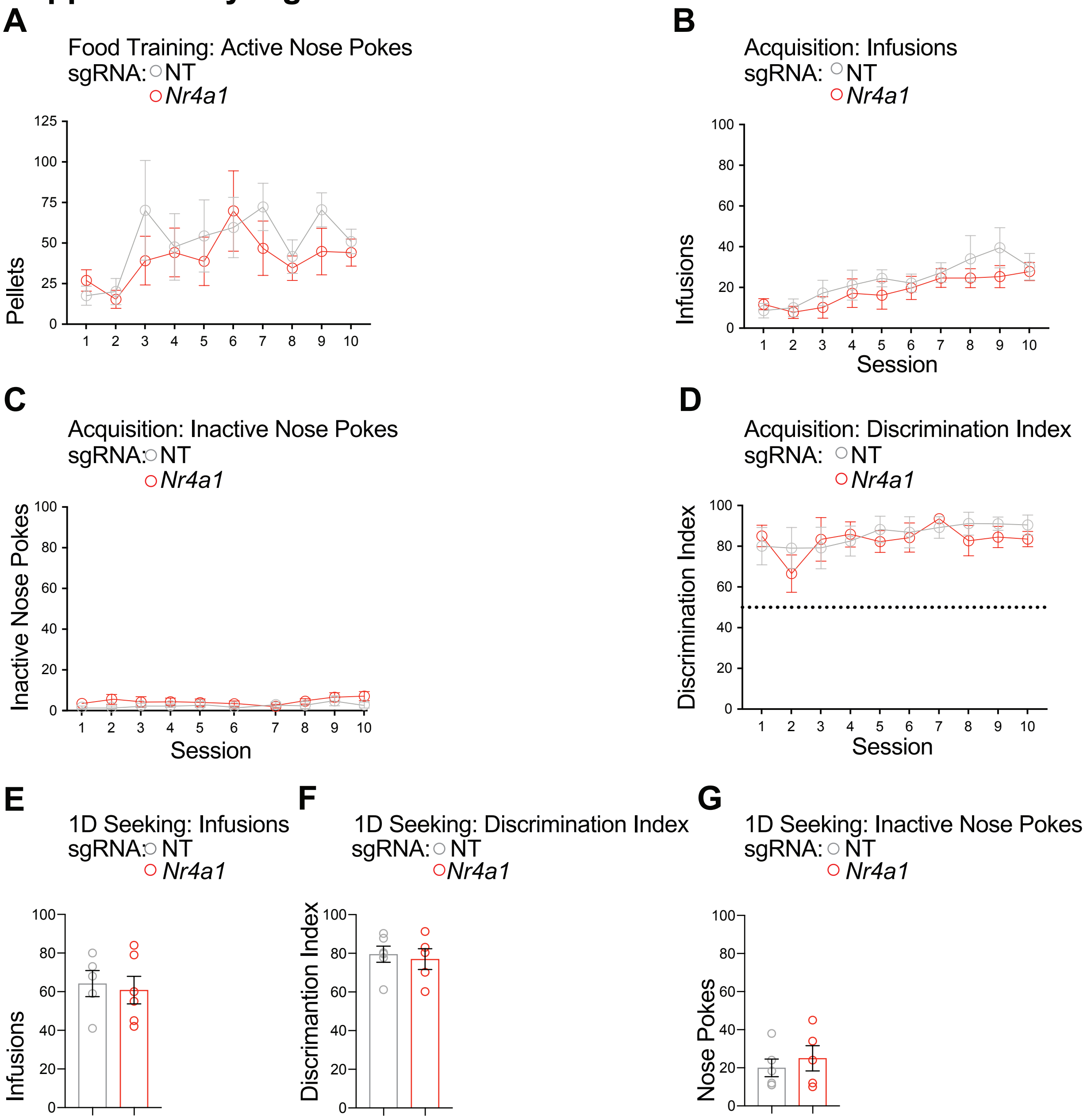

Supplementary Figure 8. CRISPR-mediated activation of Nr4a1 and cocaine SA A. No significant effect of treatment on food training prior to transfection and cocaine SA (n=5-6 mice/group, two-way repeated measures ANOVA, *P<0.05. **B.** No significant effect of treatment on infusions following CRISPR-mediated Nr4a1 activation (n=5-6 mice/group, two-way repeated measures ANOVA *P<0.05. **C.** No significant effect of treatment on inactive nose pokes (n=5-6 mice/group, two-way repeated measures ANOVA, *P<0.05. **D.** No significant effect of treatment on the discrimination index (n=5-6 mice/group, two-way repeated measures ANOVA, *P<0.05. **E.** No significant effect of treatment on infusions (saline) on 1-day seeking test (n=5-6, unpaired two-tailed t test, P=0.742). *P<0.05. F. No significant effect of treatment on the discrimination on 1-day seeking test (n=5-6, unpaired two-tailed t test, P=0.717).*P<0.05. **G.** No significant effect of treatment on inactive nose pokes on 1-day seeking (n=5-6, unpaired two-tailed t test, P=0.466).Source data and statistics provided as a Source Data file.

Supplementary Figure 9

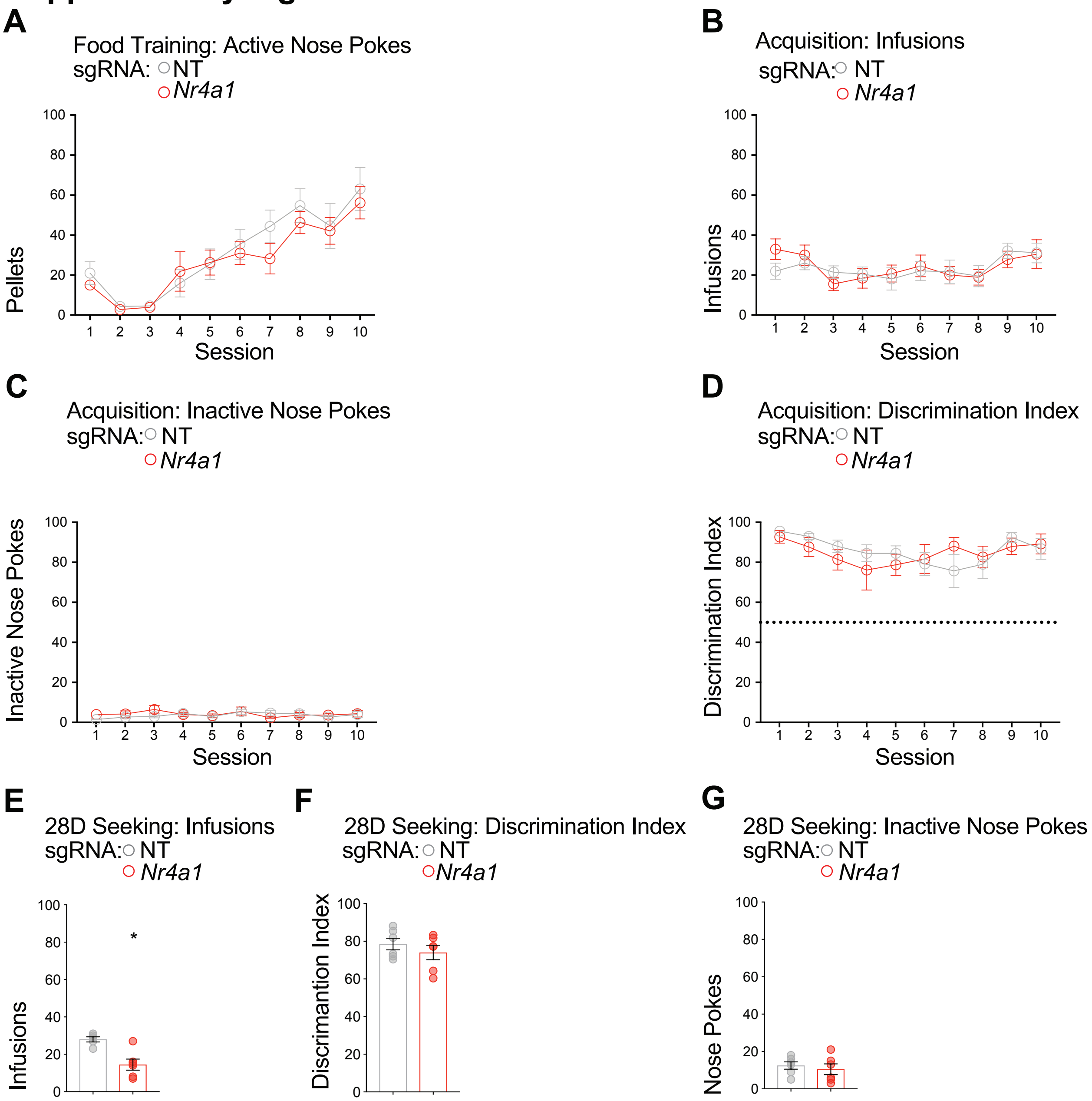

Supplementary Figure 9. CRISPR-mediated activation of *Nr4a1* and cocaine seeking A. No significant effect of treatment on in food training prior to transfection and cocaineSA (n=6 mice/group, two-way repeated measures ANOVA,*P<0.05. **B.** No significant effect of treatment on infusions during acquisition of cocaine SA (n=6 mice/group, two-way repeated measures ANOVA, *P<0.05. **C.** No significant effect of treatment on inactive nose pokes during acquisition of cocaine SA (n=6 mice/group, two-way repeated measures ANOVA, *P<0.05. **D.** No significant effect of treatment on the discrimination index during acquisition of cocaine SA (n=6 mice/group, two-way repeated measures ANOVA, *P<0.05. **E.** CRISPR-mediated Nr4a1 activation attenuated cocaine-seeking at 28-days of abstinence measured by the number of saline infusions (n=6, unpaired two-tailed t test, P=0.0037). *P<0.05. **F.** No significant effect of treatment on discrimination index on the 28-day seeking test (n=6, unpaired two-tailed t test, P=0.3834). *P<0.05. **G.** No significant effect of treatment on inactive nose pokes on 28-day seeking test f(n=6, unpaired two-tailed t test, P=0.5749). error bars represent s.e.m. Source data and statistics provided as a Source Data file.

Gene	Forward Primer	Reverse Primer
Nr4a1	AGCTTGGGTGTTGATGTTCC	AATGCGATTCTGCAGCTCTT
Cartpt	ACGAGAAGGAGCTGATCGAA	TCTCTGAGGGGAACGCAAAC
Per2	TTGGTGTGTGGGTTGTTGTG	CTACCTGGTCAAGGTGCAAGAG
Cbl	TGCTGGAAGCTCATGGACAA	CAAGCGTCTCCATCTTCCCC
Ccdc141	GTTCTTTCCTTTTGGCCGGG	TTGCCACACTTGACAACGGC
Mertk	GGGACGTTGGTGGATACGTG	CTCTTCCCACTTCTCGGCAG
Kirrel2	CTCCTCGTTTTCCTTTGCTG	ACCACCCTGGAAGGTCTCTT
Fmo2	AGGCTCCATCTTCCCAACCGTA	CCGGGTCTTTAAGGGTTTCAGG
Raver2	ATTTGGCAAGTGTGCTACCC	TCGATGGATGGAGAATAGGC

Supplementary Table 1. Primer sequences used for qPCR.

Promoter Region	Forward Primer	Reverse Primer
Nr4a1	ATTTACAACACCCCCTCCTCC	TTCCATTGACGCAGGGAGCG
Cartpt	ACACAAGAGCCGTCAATTCCA	TCGAGTTCCCAACACCGC
Per2	CACCAGCAGCTCAGTTTGTTAG	GGTTTGAGGCTGACAGAGAA
Cbl	AGTACATATTCAGTTCCCAGA	CAGACTAAGTCACATAAAGGTT

Supplementary Table 2. Primer sequences used for ChIP-qPCR.

sgRNA	Protospacer sequence 5'-3' (20-1)	PAM
Name	Note: NT 20 is converted to G in GBLOCK	
Nr4a1-366	GGGGTCCGAAATAACCGACC	AGG
Nr4a1_-231	ACGCGGGGTTCCATTGACGC	AGG
Nr4a1_-39	CTTAAGCGCTCCGTGACGCA	TGG
Nr4a1_82	ACGCGGGACCAGGCTGCGAC	TGG
Nr4a1_200	GGGTGTACGCGCGGGCGAAA	AGG

Supplementary Table 3. sgRNA sequences used for CRISPR studies.
