# Supplementary material for: Nr4a1 suppresses cocaine-induced behavior via epigenetic regulation of homeostatic target genes
Source: Nat Commun. 2020 Jan 24;11:504. doi: 10.1038/s41467-020-14331-y (PMC6981219; doi:10.1038/s41467-020-14331-y)
Supplement: Supplementary file 3 — Reporting Summary [file 41467_2020_14331_MOESM3_ESM.pdf]

nature research

Corresponding author(s): Elizabeth A Heller

Last updated by author(s): 2019 - 12-11

Reporting Summary

Nature Research wishes to improve the reproducibility of the work that we publish. This form provides structure for consistency and transparency in reporting. For further information on Nature Research policies, see Authors & Referees and the Editorial Policy Checklist.

Statistics

For all statistical analyses, confirm that the following items are present in the figure legend, table legend, main text, or Methods section.

n/a	Confirmed	
☐	☒	The exact sample size (*n*) for each experimental group/condition, given as a discrete number and unit of measurement
☐	☒	A statement on whether measurements were taken from distinct samples or whether the same sample was measured repeatedly
☐	☒	The statistical test(s) used AND whether they are one- or two-sided<br>*Only common tests should be described solely by name; describe more complex techniques in the Methods section.*
☒	☐	A description of all covariates tested
☐	☒	A description of any assumptions or corrections, such as tests of normality and adjustment for multiple comparisons
☐	☒	A full description of the statistical parameters including central tendency (e.g. means) or other basic estimates (e.g. regression coefficient) AND variation (e.g. standard deviation) or associated estimates of uncertainty (e.g. confidence intervals)
☐	☒	For null hypothesis testing, the test statistic (e.g. *F*, *t*, *r*) with confidence intervals, effect sizes, degrees of freedom and *P* value noted<br>*Give P values as exact values whenever suitable.*
☒	☐	For Bayesian analysis, information on the choice of priors and Markov chain Monte Carlo settings
☒	☐	For hierarchical and complex designs, identification of the appropriate level for tests and full reporting of outcomes
☒	☐	Estimates of effect sizes (e.g. Cohen's *d*, Pearson's *r*), indicating how they were calculated

Our web collection on statistics for biologists contains articles on many of the points above.

Software and code

Policy information about availability of computer code

Data collection

RNA-seq is collected by Hiseq4000 (illumina). Animal self-administration behavioral data was collected by Med-PC® V Software Suite (SOF-736). Animal conditioned place preference behavioral data was collected by HDV-5052 HDMI 1920x1080p Full HD Wifi Digital Video Camera (Hausbell). qPCR and qChIP data was collected by ABI 7900HT Real Time PCR Machine. Tissue was collected under a fluorescence stereoscope (Leica). For IHC experiments brain sections were imaged using as z-stacks using the 40x objective of a Zeiss LSM 810 confocal microscope (Carl Zeiss).

Data analysis

The appropriate statistical test was determined based on the number of comparisons being done. Student's t tests were used for comparison of two groups, in the analysis of qRT-PCR following in vivo CRISPR-mediated gene activation and Two-way ANOVAs were used for comparison of saline and cocaine treatment across abstinence time points; when appropriate, a post-hoc test followed to determine significant differences across multiple comparisons, in the analysis of qRT-PCR and qChIP data. One-way ANOVA was used for analysis of three or more experimental groups, when appropriate, a post-hoc test followed to determine significant differences across multiple comparisons, in the analysis of CRISPR-mediated gene regulation when compared to controls (qRT-PCR). Repeated measure two-way ANOVA was used for comparison of two groups on different observations, when appropriate, a post-hoc test followed to determine significant differences across multiple comparisons, in the analysis of cocaine self-administration and condition place preference. Main and interaction effects were considered significant at $P < 0.05$. P values greater than 0.05 and below 0.1 were considered trends. Data are expressed as mean [+ or -] s.e.m. The Grubbs test was used when appropriate to identify outliers. F tests of variance were conducted on all data sets to ensure that the data followed a normal distribution. All experiments were carried out one to three times, and data replication was observed in instances of repeated experiments. Experimental sample sizes were determined using G*power using preliminary data. Details on each statistical test can be found in the source data file.
RNA-seq: RNA-seq reads were aligned against mouse reference genome (mm9, ensemble annotation) using STAR (Version 2.4.1d) with default parameters. Aligned reads were normalized using different methods including TMM, TMM_CPM, RPKM, EDAseq_UQ, FullQuantile, Median, PoissonSeq, RUV 94 and PORT (https://github.com/itmat/Normalization). Differential gene expression analysis was performed using different models: DEseq2-Wald, EdgeR-Robust-LRT, LimmaVoom, MannWhitney and Parametric t-test. A gene is identified as differential expressed gene if it is detected by the combination of 5 different methods.
Animal behavioral data was analyzed using ANY-Maze (version 4.99).

Gene ontology enrichment analysis for differentially expressed genes was performed using the biological process annotation tool in DAVID Bioinformatics Resources (version 6.8).
Other data analysis that are include in this study were used Prism 8 (GraphPad)

For manuscripts utilizing custom algorithms or software that are central to the research but not yet described in published literature, software must be made available to editors/reviewers. We strongly encourage code deposition in a community repository (e.g. GitHub). See the Nature Research guidelines for submitting code & software for further information.

Data

Policy information about availability of data

All manuscripts must include a data availability statement. This statement should provide the following information, where applicable:

- Accession codes, unique identifiers, or web links for publicly available datasets
- A list of figures that have associated raw data
- A description of any restrictions on data availability

Data Availability
Source data presented in Fig. 1 and 2, Supplementary Fig. 2 and 4 can be accessed through GEO upon publication using accession number GSE141520. All data are reported in main text and in supplementary data tables.

Field-specific reporting

Please select the one below that is the best fit for your research. If you are not sure, read the appropriate sections before making your selection.

☒ Life sciences ☐ Behavioural & social sciences ☐ Ecological, evolutionary & environmental sciences

For a reference copy of the document with all sections, see nature.com/documents/nr-reporting-summary-flat.pdf

Life sciences study design

All studies must disclose on these points even when the disclosure is negative.

Sample size	Sample sizes for behavioral experiments were determined by the current standard used for mice in behavioral neuroscience experiments, based on the minimal amount of mice required to detect significance. In addition, the software g*Power was used to For imaging experiments, between 3 and 5 mice were imaged per experimental paradigm. For molecular assays, n = 5 was used as a minimum. For Chip experiments bilateral 2mm punches were dissected from NAc of a single mouse; n =6 per treatment, as described in methods. For in vivo CRISPR Chip experiments unilateral 1.2 mm punches were dissected from NAc, 3 mice were pooled for an n of 1, n = 6 per treatment, as described in methods.
Data exclusions	For mouse self-administration acquisition was defined >10 responses for 3 consecutive days. For conditioned place preference experiments mice were excluded if they showed a significant initial bias of greater that 35% during pre-test. For in vivo CRISPR transfections, mice were excluded for off targeting visualized under stereoscope.
Replication	RNA-seq findings were validated and replicated via qPCR in a separate cohort of mice. In vivo and in vitro CRISPR studies were replicated three times. qPCR studies were replicated twice. All replication was successful
Randomization	Mice were allocated into groups by cage. To help control for variation, all samples for each individual assay were processed by the same person.
Blinding	For all behavioral experiments experimenter was blinded to treatments. For all molecular experiments samples were coded and experimenter was blinded.

Reporting for specific materials, systems and methods

We require information from authors about some types of materials, experimental systems and methods used in many studies. Here, indicate whether each material, system or method listed is relevant to your study. If you are not sure if a list item applies to your research, read the appropriate section before selecting a response.

Materials & experimental systems

n/a	Involved in the study	
☐	☒	Antibodies
☐	☒	Eukaryotic cell lines
☒	☐	Palaeontology
☐	☒	Animals and other organisms
☒	☐	Human research participants
☒	☐	Clinical data

Methods

n/a	Involved in the study	
☒	☐	ChIP-seq
☒	☐	Flow cytometry
☒	☐	MRI-based neuroimaging

Antibodies

Antibodies used	H3K4me3100 (EMD Millipore 07-473) Manufacturer validation http://www.emdmillipore.com/US/en/product/Anti-trimethyl-Histone-H3-Lys4-Antibody,MM_NF-07-473; ChIP in brain citation: DOI: 10.1016/j.cell.2017.09.047<br>H3K27me3 (EMD Millipore 07-449) Manufacturer validation http://www.emdmillipore.com/US/en/product/Anti-trimethyl-Histone-H3-Lys27-Antibody,MM_NF-07-449; ChIP in brain citation: DOI: 10.1016/j.celrep.2017.07.046<br>H3K27ac (EMD Millipore 07-360) Manufacturer validation http://www.emdmillipore.com/US/en/product/Anti-acetyl-Histone-H3-Lys27-Antibody,MM_NF-07-360; ChIP in brain citation: DOI: 10.1093/nar/gkv589<br>Nr4a1 (Novus NB100-56745), Manufacturer validation https://www.novusbio.com/products/ngfi-b-alpha-nur77-nr4a1-antibody_nb100-56745 ChIP DOI:10.1158/1541-7786.MCR-08-0473<br>IgG (Novus NBP2-24891) Manufacturer validation;
Validation	Nr4a1 antibody were selected from Novus Biologicals. We validated this antipbody using IgG for ChIP experiment (Supp Fig. 5). In has been validated in ChIP in DOI:10.1158/1541-7786.MCR-08-0473; ChIP: Fig 5 (Panc1 cells) Note: Nur77 was detected in the nuclear, but not the cytoplasmic fraction of Panc1 cells by WB (Fig 6D). Note: The Nur77 IMG-528 antibody was Nur77 siRNA transfected validated by western blot. Nur77 siRNA, but not scrambled siRNA reduced the intensity of the WB signal in PANC1 and L3.6pi cells (Figs 3C, 4B).

Eukaryotic cell lines

Policy information about cell lines

Cell line source(s)	Neuro-2a (ATCC® CCL-131™) ATCC
Authentication	Authentication was performed by ATCC
Mycoplasma contamination	All cell lines tested negative for mycoplasma contamination
Commonly misidentified lines (See ICLAC register)	No commonly misidentified lines were used

Animals and other organisms

Policy information about studies involving animals; ARRIVE guidelines recommended for reporting animal research

Laboratory animals	Male and female mice were used, 8-10 week-old C57BL/6J mice
Wild animals	N/A
Field-collected samples	N/A
Ethics oversight	Protocols approved by the Institutional Animal Care and Use Committee of the University of Pennsylvania

Note that full information on the approval of the study protocol must also be provided in the manuscript.
