# Supplementary material for: Nr4a1 suppresses cocaine-induced behavior via epigenetic regulation of homeostatic target genes
Source: Nat Commun. 2020 Jan 24;11:504. doi: 10.1038/s41467-020-14331-y (PMC6981219; doi:10.1038/s41467-020-14331-y)
Supplement: Supplementary file 4 — Description of Additional Supplementary Files [file 41467_2020_14331_MOESM4_ESM.pdf]

Description of Additional Supplementary Files

File Name: Supplementary Data 1

Description: Supplementary Data File 1. RNA-Seq results NAc, VTA and PFC
